# Tumor-induced loss of mural Connexin 43 gap junction activity promotes endothelial proliferation

**DOI:** 10.1186/s12885-015-1420-9

**Published:** 2015-05-23

**Authors:** Mayur Choudhary, Christine Naczki, Wenhong Chen, Keith D. Barlow, L. Douglas Case, Linda J. Metheny-Barlow

**Affiliations:** 1 Department of Radiation Oncology, Wake Forest School of Medicine, Medical Center Boulevard, Winston-Salem, NC 27157 USA; 2 Department of Cancer Biology, Wake Forest School of Medicine, Medical Center Boulevard, Winston-Salem, NC 27157 USA; 3 Department of Biostatistical Sciences, Wake Forest School of Medicine, Medical Center Boulevard, Winston-Salem, NC 27157 USA; 4 Wake Forest Comprehensive Cancer Center, Wake Forest School of Medicine, Medical Center Boulevard, Winston-Salem, NC 27157 USA; 5 Current address: Duke Eye Center, 2351 Erwin Road, AERI Room 4000, Durham, NC 27705 USA

**Keywords:** Breast cancer, Connexin 43, Gap junction communication, Pericyte, Mural cell, Endothelial cell, Angiogenesis

## Abstract

**Background:**

Proper functional association between mural cells and endothelial cells (EC) causes EC of blood vessels to become quiescent. Mural cells on tumor vessels exhibit decreased attachment to EC, which allows vessels to be unstable and proliferative. The mechanisms by which tumors prevent proper association between mural cells and EC are not well understood. Since gap junctions (GJ) play an important role in cell-cell contact and communication, we investigated whether loss of GJ plays a role in tumor-induced mural cell dissociation.

**Methods:**

Mural cell regulation of endothelial proliferation was assessed by direct co-culture assays of fluorescently labeled cells quantified by flow cytometry or plate reader. Gap junction function was assessed by parachute assay. Connexin 43 (Cx43) protein in mural cells exposed to conditioned media from cancer cells was assessed by Western and confocal microscopy; mRNA levels were assessed by quantitative real-time PCR. Expression vectors or siRNA were utilized to overexpress or knock down Cx43. Tumor growth and angiogenesis was assessed in mouse hosts deficient for Cx43.

**Results:**

Using parachute dye transfer assay, we demonstrate that media conditioned by MDA-MB-231 breast cancer cells diminishes GJ communication between mural cells (vascular smooth muscle cells, vSMC) and EC. Both protein and mRNA of the GJ component Connexin 43 (Cx43) are downregulated in mural cells by tumor-conditioned media; media from non-tumorigenic MCF10A cells had no effect. Loss of GJ communication by Cx43 siRNA knockdown, treatment with blocking peptide, or exposure to tumor-conditioned media diminishes the ability of mural cells to inhibit EC proliferation in co-culture assays, while overexpression of Cx43 in vSMC restores GJ and endothelial inhibition. Breast tumor cells implanted into mice heterozygous for Cx43 show no changes in tumor growth, but exhibit significantly increased tumor vascularization determined by CD31 staining, along with decreased mural cell support detected by NG2 staining.

**Conclusions:**

Our data indicate that i) functional Cx43 is required for mural cell-induced endothelial quiescence, and ii) downregulation of Cx43 GJ by tumors frees endothelium to respond to angiogenic cues. These data define a novel and important role for maintained Cx43 function in regulation of vessel quiescence, and suggest its loss may contribute to pathological tumor angiogenesis.

**Electronic supplementary material:**

The online version of this article (doi:10.1186/s12885-015-1420-9) contains supplementary material, which is available to authorized users.

## Background

A functional blood vessel is composed of the endothelium, the cell layer that forms the channel through which blood flows, and mural cells, the vascular smooth muscle cells (vSMC) and pericytes that serve to support and stabilize the endothelium. Proper physical and functional association between mural cells and endothelial cells (EC) causes EC to become quiescent [1]. In addition, mural cells contribute to vessel stability via regulation of deposition of basement membrane proteins and enhancement of tight junctions between endothelial cells [2, 3]. In contrast, the vasculature formed by a tumor is highly disorganized and exhibits decreased and abnormal association with mural cells [4, 5], which allows the vessel to be unstable, leaky and proliferative. However, the molecular determinants that mediate the inhibitory interactions between mural cells and endothelial cells, and the mechanism(s) by which tumors prevent proper association between mural cells and the endothelium, have not been well elucidated. Knowledge of the mechanism(s) by which mural cells dissociate from vessels would significantly increase the understanding of physiological blood vessel development, as well as pathological conditions such as tumor angiogenesis in which these basic processes appear to be deregulated.

Gap junctions (GJ) are membrane channels that allow the transfer of ions, second messengers, and other small molecules between adjacent cells [6]. GJ are composed of connexin proteins, of which the product of the GJA1 gene, Connexin 43, is a main component in the vasculature. Functional heterologous gap junctions have been described between EC and mural cells [7–9]. Indeed, Cx43-based gap junctions have been shown to be required for the endothelial-induced differentiation of mural cell precursors during vessel assembly [9]. In addition, these gap junctions play a role in integrating vasopressive signals along the vessel to maintain vascular tone [10] and are required for proper coronary vessel patterning [11, 12]. In this study, we have explored the role of Cx43 in the inhibitory interactions between mural cells and endothelial cells and the alteration of Cx43 expression and function in a model of tumor angiogenesis. We find that Cx43-based gap junction activity is required for the mural cell-induced endothelial quiescence that is characteristic of stabilized vessels, and that breast tumor cell downregulation of mural cell Cx43 is sufficient to release endothelial cells from this inhibition to allow endothelial proliferation. Further, we show that tumor angiogenesis is increased in a Cx43-deficient host. These data therefore provide a possible mechanistic explanation for the maturation defect characteristic of vasculature in tumors and other angiogenic pathologies which allows vessels to be proliferative.

## Methods

### Cell culture and reagents

MDA-MB-231 human breast cancer cells were obtained from the Georgetown University Repository and maintained in IMEM, 10 % FBS. STR analysis (CellCheck, RADIL, University of Missouri) confirmed origin of these cells as MDA-MB-231. Conditioned media (CM) was prepared by incubating a 90 % confluent monolayer of MDA-MB-231 cells in EBM-2 (Lonza, Walkersville, MD) for 24 h. Human MCF10A and MCF7 from the WFU Tissue Virus and Vector Core were maintained in RPMI-1640 + 10 % FBS. To screen for relative potency of conditioned media, flasks of ~90 % confluent cells were incubated with EBM-2 for 24 h. In some experiments, cells from the conditioning flasks were then trypsinized and counted, and all media equalized to the same cell number/ml media. vSMC (primary human coronary artery smooth muscle cells, passage 5 to 8, Lonza [5 lots] or Cascade Biosciences, Eugene, OR [1 lot]) and HBVP (human brain vascular pericytes, passage 4–8, ScienCell, Carlsbad, CA) were maintained in SmGM-2 (Lonza). HUVEC (passage 5 to 9, Lonza), GFP-HUVEC (passage 4 to 6, Angio-Proteomie, Boston, MA), and GFP-HBMEC (human brain microvascular pericytes, passage 4–7, Angio-Proteomie) were maintained in complete EGM2-MV (Lonza). C3H10T1/2 (passage 12 to 14) and MDA-MB-468 cells were purchased from ATCC (Manassas, VA) and maintained in low glucose DMEM (Hyclone, South Logan, UT), 10%FBS. Eo771 mouse mammary tumor cells (kind gift of Dr. Francis Sirotnak, Memorial Sloan Kettering Cancer Center) were maintained in RPMI-1640 + 10 % FBS. All incubations were carried out in a humidified atmosphere at 37 °C and 5 % CO_2_.

### Measurement of endothelial proliferation in Co-culture

Three co-culture cell models were used: (i) *vSMC and HUVEC:* For tumor conditioned media experiments, GFP-HUVEC (1200–1800 cells/well of 96 well plate) were co-cultured with vSMC at a ratio of 1:1.5 in EGM2-MV for 24 h, followed by addition of Mock and MDA-MB-231 CM supplemented with 1 % FBS. GFP fluorescence (Exc 485 nm, Em 520) was measured on a BMG Labtek Fluorostar Optima plate reader on day 4 as a measure of HUVEC cell number. GFP-HUVEC and vSMC monocultures plated in Mock and MDA-MB-231 CM were used as controls. For Cx43 overexpression, vSMC were nucleofected with control or pCMV6-XL5-Cx43 vector twenty-four hours prior to plating in co-culture and analyzed as above. For knockdown experiments, PKH26-labeled vSMC nucleofected with non-targeting siRNA or siRNA specific for Cx43 were co-cultured with GFP-HUVEC or in monoculture in 6-well plates. On indicated day, cells were trypsinized and counted on a hemocytometer followed by FACS analysis to determine relative percentage of red (vSMC) or green (HUVEC) cells in the suspension. Total cell counts from hemocytometer readings and percentage counts from FACS were used to determine number of HUVEC in the co-culture. Co-cultures were also set up in the presence of 250 μM Cx43 GAP26 (sequence VCYDKSFPISHVR) or scrambled control peptide (GenScript, Piscataway, NJ); cultures received fresh media with GAP26 peptide on the third day of culture. (ii) *HBVP and GFP-HBMEC*: GFP-HBMEC were co-cultured with HBVP at a ratio of 1:1.5 as above, except that CM was supplemented with 2.5 % FBS. (iii) *C3H10T1/2 and HUVEC:* C3H10T1/2 cells were nucleofected with non-targeting or Cx43-targeted siRNA, allowed to recover overnight, labeled with CellTracker Green, then added to PKH-26 labeled HUVEC. Controls consisted of C3H10T1/2 and HUVEC cultured alone in identical conditions. On indicated day, cells were trypsinized and quantified by FACS as above, except that red fluorescence indicated HUVEC and green indicated C3H10T1/2.

### Western blot analysis

vSMC were starved 16–18 h in basal EBM-2, 0.1 % BSA then stimulated with Mock or MDA-MB-231 CM for 24 h and lysed in RIPA buffer (1 % NP-40, 0.5 % Sodium Deoxycholate, 1 % SDS) containing 1X Thermo Scientific Halt Phosphatase Inhibitor Cocktail and Roche Complete Mini Protease Inhibitor. Protein content was quantified and equal quantity of protein separated by SDS-polyacrylamide gel electrophoresis and transferred onto nitrocellulose membrane (Thermo Scientific, Waltham, MA). After blocking, the membrane was probed with antibodies specific for Cx37 (Abcam), Cx40 (Millipore), Cx43 (Cell Signaling, Danvers, MA), Cx45 (Sigma) and loading control (Tubulin, Labvision, Fremont, CA; β-Actin, Sigma; or GAPDH, Cell Signaling), followed by exposure to appropriate horseradish-peroxidase-linked secondary antibody (Amersham Life Sciences, Piscataway, NJ). Bound antibody was detected using chemiluminescence (ECL Plus, Amersham) and quantified using ImageJ software (NIH) or Scion Image software. Data were normalized to loading control and expressed as relative Cx43 levels compared to corresponding mock.

### Nucleofection

vSMC or C3H10T1/2 cells at 80 % confluence were trypsinized and nucleofected with control non-targeting siRNA or the appropriate (human or mouse) siRNA pool directed against Cx43 (Dharmacon, Lafayette, Colorado), or pCMV6-XL5-Cx43 or control vector (Origene, Rockville, MD), using an Amaxa NucleofectorII and Primary Smooth Muscle kit solution (Lonza) per manufacturer’s directions.

### Parachute assay

GJ activity was measured by a noninvasive parachute assay as described [13] using vSMC as “donor” cells and HUVEC as “acceptor” cells. HUVEC acceptor cells were labeled with the nontransferable dye PKH-26 (Sigma-Aldrich, St Louis, MO) using the manufacturer’s protocol, washed with PBS, resuspended in fresh media and 5 × 10^5^ cells plated in 6-cm dishes. vSMC donor cells were loaded with 0.1 μΜ calcein-AM (Sigma-Aldrich) in growth medium with 2.5 mM probenecid (Biomol, Plymouth Meeting, PA) for 30 min at 37 °C. After loading, donor cells were trypsinized and 10^5^ cells dropped onto PKH-26-labeled acceptor cells and incubated at 37 °C for 3 h. For Mock and MDA-MB-231 CM groups, vSMC were treated for 24 h prior to calcein-AM loading. For Cx43 alterations, vSMC were nucleofected with siRNA or plasmid twenty-four hours prior to calcein-AM loading. The GJ communication inhibitor 18-α*-*Glycyrrhetinic acid (MP Biomedical, Irwine, CA) was used as negative control. To assess degree of dye coupling, cells were harvested in single cell suspensions by trypsin digestion, and fluorescence detected on a FACS Calibur flow cytometer (BD Biosciences, San Jose, CA) using a 488 nm filter for calcein and a 585 nm filter for PKH-26. GJ intercellular (GJIC) communication transfer ratio was calculated using the formula: (Number of acceptor cells X Fraction of double-labeled cells)/ Number of donor cells. Data are presented as Relative Transfer Ratio, which was calculated by normalizing values to Control Mock.

### Confocal imaging

GFP-HUVEC (10,000-15,000 cells/well) were co-cultured with vSMC at a ratio of 1:1.5 in EGM2-MV for 24 h, then media replaced with EBM-2 + 1 % FBS for 48 h. Cells were then stimulated with Mock or MDA-MB-231 CM treatment for 24 h. For immunolabeling, fixed cells were probed with mouse monoclonal anti-Cx43IF1 antibody (Fred Hutchison Cancer Research Center, Seattle, WA) followed by anti-mouse secondary antibody conjugated to Alexa Fluor 555 (Cell Signaling). Fluorescently labeled cells were imaged on a Zeiss LSM 510 Axiovert 100 M confocal microscope (Carl Zeiss MicroImaging Inc, Thornwood, NY) using a Exc 488 nm/Em 505 (GFP-HUVEC) and Ex543/Em650 (Alexa Fluor 555) at x200 with additional digital magnification up to x440. The 8-bit images were obtained in a 512 x 512 pixel format with 6–11 optical slices of 1.7 μm thickness each. vSMC-GFP-HUVEC cell pairs were marked and the cross-section of the z-stack was scanned across the X-Y plane using the orthogonal function of the LSM Image Examiner (Carl Zeiss Microimaging Inc.). Cx43 staining extending beyond margin of GFP-HUVEC was counted as heterotypic gap junction.

### Quantitative RT-PCR

Total RNA was extracted using TRIzol® and DNase digested using RT^2^ qPCR grade RNA Isolation kit (SA Biosciences, Frederick, MD). Synthesis of cDNA was carried out using SABiosciences RT^2^ First Strand Kit with 400–800 ng total sample RNA. PCR was performed using an ABI-PRISM 7000 Detection System thermal cycler (Applied Biosystems, Carlsbad, CA) in a 25 μl reaction consisting of SA Biosciences RT^2^ qPCR Master Mix/ROS, 1 μl human GJA1 or GAPDH primers (Real Time Primers, Elkins Park, PA) primers, and 1 μl of template cDNA using the cycling conditions: 10 min at 95 °C (1 cycle): 95 °C 15 s, 60 °C for 1 min (45 cycles). Data were normalized to GAPDH and fold-change for 231 CM-treated relative to mock calculated using the ΔΔCt method [14].

### Mouse generation and tumor implantation

All experiments were approved by the Wake Forest School of Medicine Institutional Animal Care and Use Committee. Mice heterozygous for the Cx43 gene were generated by crossing mice with the second exon of Cx43 flanked by loxP sites (Jackson Laboratories, strain #008039, [15]) with mice in which CRE is expressed ubiquitously under control of the CMV promoter (Jackson Laboratories, strain #006054), both of which were generated on a C57Bl/6 background. Related mice homozygous for the floxed Cx43 gene (which express normal levels of Cx43) were used as wild-type controls. Western analysis of mammary fat pads without tumor implantation confirmed reduction of Cx43 protein in the Cx43 Het mice to 47.3+/−10.7 % that of control mice (*n* = 8). Eo771 mouse medullary adenocarcinoma cells (2.5x10^5^), derived from a spontaneous mammary tumor in a C57Bl/6 mouse, were injected into the inguinal mammary fat pad of WT (*n* = 11) and Cx43 Het mice (*n* = 10). Tumor size was measured twice a week using a digital caliper, and tumor volume estimated using the formula (((L + W)/2)^3)/2 [16]. The experiment was terminated at 14 days post-injection.

### Immunofluorescence

Eo771 mammary fat pad tumors were removed and flash frozen in OCT medium (Tissue-Tek) and cut to 7 μm sections, allowed to thaw to room temp for 30 min, fixed for 10 min in 100 % acetone and blocked for 1 h in 3 % BSA in PBS. Sections were incubated in anti-CD31 (BD Biosciences) and/or anti-NG2 (Millipore) overnight at 4 °C followed by secondary Alexa 488 or 555 (Cell Signaling) for 1 h at room temp and mounted with Fluorogel + DAPI (EMS). Fluorescent microscopy was performed on an Olympus IX70 scope and images acquired at 20x with a Retiga 200R camera. The area of CD31 positive fluorescent pixels across five random fields was quantified using Image J software and averaged for each of 8 tumors per experimental group. To assess vessel mural cell investiture, relative area of CD31 positive vessels were quantified for area of NG2 positive pixels on 5 random vessels per tumor, 3 tumors per group and quantified using Image J software.

### Statistical analysis

Statistical analyses were performed in the Biostatistics Core Facility of the Comprehensive Cancer Center of Wake Forest University using SAS version 9.2. All data are presented as means +/− s.e.m. across experiments of the averages of the replicate values within an experiment. The significance of Cx43 knockdown/inhibition or tumor CM treatment on dye transfer or endothelial proliferation was determined using a mixed model ANOVA with linear contrasts to assess specific pairwise comparisons between groups of interest. Experimental conditions were assumed to be nested within experiments, and the Tukey-Kramer method was used to adjust for multiple contrasts. Tumor growth over time was analyzed using repeated measures ANOVA with square root transformed data to improve normality and stabilize variances. Student’s t-test was used to compare gap junction quantification by microscopy, CD31 area, NG2/CD31 ratio and tumor mass; for CD31 area, data were log transformed to normalize the data. Statistical significance was set at p < 0.05.

## Results

### Breast tumor cells release endothelial cells from mural cell growth inhibition

**Fig. 1:**
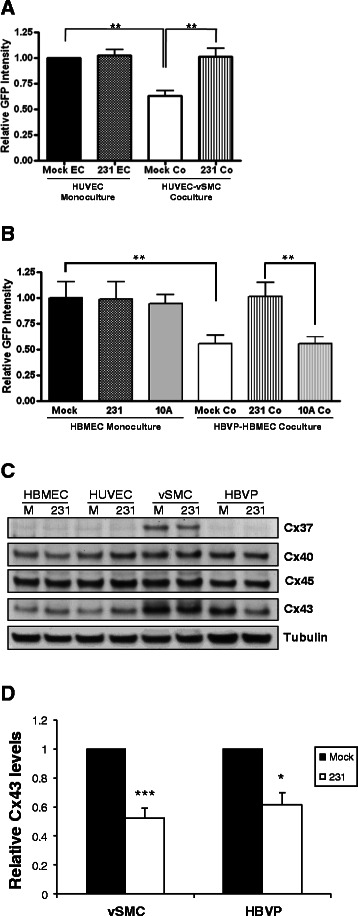
Breast tumors release endothelial cells from mural cell inhibition and downregulate mural cell Cx43. vSMC and GFP-HUVEC **a** or HBVP and GFP-HBMEC **b** were plated alone or in co-culture overnight in normal growth media, then cultured in control media (‘Mock’) or media conditioned by MDA-MB-231 cells (‘231’) or MCF10A cells (‘10A’). At the end of four days, fluorescence was quantified. Data shown are representative of three experiments performed in duplicate or triplicate (**p < 0.01; ***p = 0.001). **c** Western analysis of indicated endothelial or mural cells starved overnight then stimulated for 24 h with control media (‘Mock’) or media conditioned by tumor cells (‘231’) and are representative of at least 2–4 replicate experiments for each cell line. **d** Quantification of Cx43 protein levels compared in vSMC and HBVP treated for 24 h with media conditioned by MDA-MB-231 tumor cells or control mock media normalized to tubulin levels (*n* = 6 different vSMC donor lots; *** *p* < 0.001; for HBVP *n* = 3 independent experiments with 2 different donor lots; *p < 0.05)

The ability of mural cells to inhibit the proliferation of endothelial cells is well established [1, 17, 18]. In order to study the effects of tumors on mural cell dissociation at the initiation of angiogenesis, we first tested whether tumors reverse the inhibitory effects of mural cells in an *in vitro* endothelial cell-mural cell co-culture using primary human vSMC as a mural cell model and GFP-labeled HUVEC as the endothelial component. As seen in Fig. 1a, media conditioned by tumor cells had minimal effect on the proliferation of endothelial cells cultured alone compared to mock conditioned media after four days in culture (*p* = 0.903, ns). Consistent with previous reports [17], endothelial cell proliferation was decreased in the presence of vSMC when co-cultured in control media (decreased 39.6+/−4.9 % from control monoculture, p < 0.01). In contrast, when co-cultures were first allowed to associate overnight then exposed for four days to media conditioned by MDA-MB-231 breast tumor cells, the ability of mural cells to inhibit endothelial proliferation was completely reversed. The ability of tumor conditioned media to override mural cell inhibition of endothelial proliferation was also observed using a co-culture of microvascular pericytes and microvascular endothelial cells (Fig. 1b, 44.7+/−3.2 % decrease from control monoculture, *p* = 0.002). In contrast, media conditioned by nontumorigenic MCF10A mammary epithelial cells did not promote endothelial proliferation in co-culture. The tumor-induced loss of the ability of mural cells to inhibit endothelial proliferation was unlikely due to a decrease in mural cell number following exposure to tumor conditioned media, as tumor conditioned media stimulates proliferation of mural cells (Additional file 1: Figure S1). This co-culture system demonstrates tumor-dependent subversion of stabilizing mural cell-endothelial cell interactions and provides a means to interrogate the mechanisms by which tumors disrupt inhibitory signals required for this effect.

### Breast tumor cells downregulate Cx43 protein in mural cells but not endothelial cells

Since mural cell mediated inhibition of endothelial proliferation is reportedly contact-dependent [17], we analyzed expression of gap junctional molecules in endothelial cells and mural cells following exposure to media conditioned by breast cancer cells. While no consistent decreases in connexin expression in response to MDA-MB-231 tumor cell conditioned media was observed in either endothelial cell line, tumor conditioned media decreased total protein levels of Cx43 in both mural cell types after 24 h treatment (Fig. 1c). Although a tumor-induced decrease in Cx37 in vSMC was also detected, previous reports indicate that heterocellular junctions between vascular endothelial cells and mural cells are composed primarily of mural cell Cx43 [9] and endothelial Cx43/40 [19], with limited presence of Cx37 at heterocellular junctions in the microvasculature [19, 20]. We therefore elected to further pursue the role of Cx43 regulation in mural cells. The ability of MDA-MB-231 cell conditioned media to decrease Cx43 protein levels was consistently observed in human vSMC isolated from at least 6 out of 7 different donors and HBVP from at least two different donors, with Cx43 protein levels reduced 48+/−7.1 % and 39+/− 9.0 % (Fig. 1d) in responding cells, respectively.

### Breast tumor conditioned media inhibits Cx43-based GJ Communication between EC and MC

**Fig. 2:**
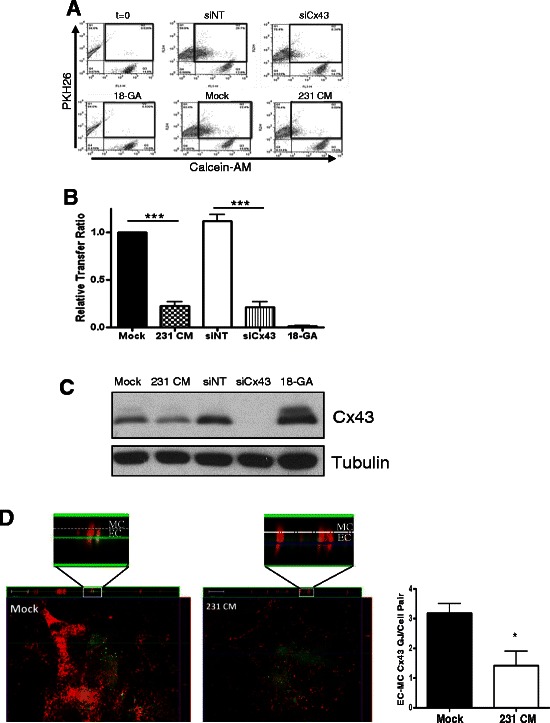
Gap junction communication between mural cells and endothelial cells requires mural cell Cx43 and is decreased by tumor cells. Untransfected vSMC or vSMC nucleofected with non-targeting (‘siNT’) or Connexin43-specific (‘siCx43’) siRNA were treated with control (‘Mock’) or media conditioned by MDA-MB-231 cells (‘231 CM’), loaded with calcein-AM, and plated onto PKH26-labeled HUVEC. Dye transfer from vSMC to HUVEC was quantified using flow cytometry. **a**. Representative flow cytometry dot plots showing dye transfer as percentage of double-labeled cells in upper right quadrant. **b**. Ratio of dye transfer as calculated in Materials and Methods. Data represent the mean of four to five independent experiments performed in triplicate +/−s.e.m. (duplicate for 18-GA); ***p < 0.001. **c**. Western analysis of Connexin 43 in vSMC samples incubated in parallel with experiment from A. **d**. vSMC and GFP-HUVEC were plated in co-culture for 72 h as in ‘Methods’, then stimulated for 24 h with control media (‘Mock’) or media conditioned by MDA-MB-231 cells (‘231 CM’), and the number of heterocellular Cx43-expressing junctions (red) extending between endothelial cells (green) and mural cells quantified by confocal Z-stack analysis. Top panel, representative confocal images; inset, magnification of z-stack image of Cx43 gap junction. Bottom panel, quantification of Cx43-positive heterotypic gap junctions as seen in top panel. Data represent the quantification from paired cells over three experiments (*n* = 46–48 cell pairs each condition)+/−s.e.m. (*p < 0.05)

We next addressed whether breast tumor cell-induced loss of vSMC Cx43 affected heterotypic GJ communication between vSMC and endothelial cells using a parachute dye transfer assay quantified by flow cytometry. In agreement with previous studies [21], significant dye transfer was observed between mural cells and endothelial cells (Fig. 2a, b). In contrast, vSMC exposed to breast tumor conditioned media demonstrated a significant decrease (to 22.3+/−5.0 % of control) in capacity to transfer dye to endothelial cells (Fig. 2b), suggesting that tumor media prevents the formation of functional heterocellular gap junctions. Similarly, knock down of Cx43 by siRNA also nearly completely abolished the transfer of dye from vSMC to endothelial cells (down to 21.2+/−5.1 % of control) indicating that Cx43 is the main mural cell connexin protein mediating GJ communication with endothelial cells. Lysates from vSMC monocultures plated in parallel probed for total Cx43 confirm the magnitude of protein loss in response to the indicated treatment (Fig. 2c). The chemical GJ inhibitor 18-α-glycyrretinic acid, which does not affect Cx43 levels, nearly completely abolishes the transfer (Fig. 2b, c), demonstrating that observed dye transfer is mediated by GJ. Consistent with this, confocal detection of Cx43 gap junctions extending between mural cells and endothelial cells in co-culture, which seemed to preferentially form when mural cells oriented themselves over endothelial cells, was quantifiably decreased following treatment with tumor conditioned media (3.19+/−0.32 mock vs. 1.43+/−0.47 with 231 conditioned media, *p* = 0.01, Fig. 2d). Together these data indicate that tumor cells dowregulate the major mural cell connexin mediating GJ transfer between mural cells and endothelial cells to disrupt established heterotypic GJ communication.

### Regulation of Cx43 by tumor cells

**Fig. 3:**
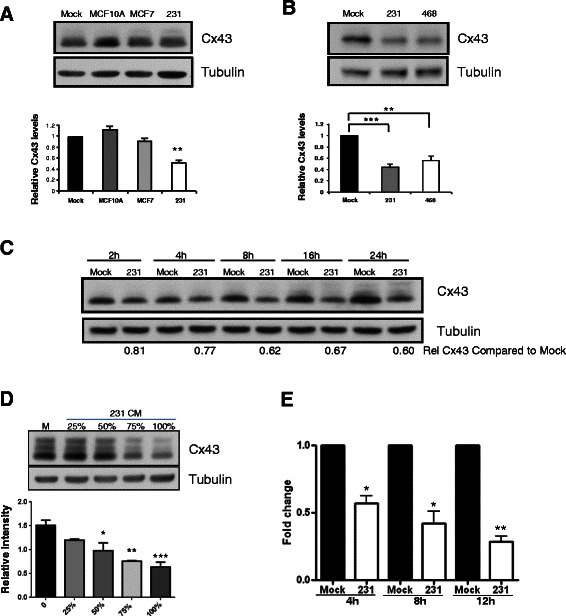
Breast tumor downregulation of Cx43 in vSMC is time- and dose- dependent. **a**, **b**. vSMC were stimulated for 24 h with media conditioned by the indicated cell line and lysates analyzed for Cx43 levels. Bottom panels represent quantification of **a***n* = 4 experiments, **b***n* = 5 experiments, *p < 0.05; **p < 0.01; ***p < 0.001. **c**. vSMC were treated for the indicated time with media conditioned by MDA-MB-231 cells and lysates analyzed by Western. Data representative of two to four experiments. **d** vSMC were treated for 24 h with media conditioned by varying dilutions of MDA-MB-231 cell CM. Bottom panel represents quantification from 3 independent experiments. **e**. mRNA was isolated and subjected to quantitative PCR analysis. Data were normalized to GAPDH levels and fold change compared to Mock; data represent the mean of three independent experiments each performed in technical triplicate (*p < 0.05; **p < 0.01)

In order to determine whether cells other than MDA-MB-231 possess the ability to downregulate Cx43 in mural cells, we screened conditioned media from a panel of human breast cancer cell lines. Consistent with its inability to release endothelial cells from mural cell inhibition, media from immortalized non-tumorigenic mammary epithelial cell line MCF10A did not alter Cx43 levels in vSMC after 24 h treatment (Fig. 3a). Similarly, media from less aggressive estrogen receptor positive (ER+) cells such as MCF7 also did not consistently diminish Cx43 levels, whereas media from more highly aggressive ER- cells such as the MDA-MB-468 cells decreased Cx43 protein levels more similar to that seen with MDA-MB-231 CM (45 % and 56 % respectively) (Fig. 3b).

Mechanistically, we determined that the downregulation of Cx43 protein by MDA-MB-231 tumor cells is time- and dose-dependent (Fig. 3c, d). Although small changes in Cx43 protein levels may be evident at shorter time points (2 h, relative Cx43 protein 0.88 compared to 2 h mock, *n* = 3, *p* = 0.16, ns; 4-5 h, relative Cx43 protein 0.78, *n* = 4. *p* = 0.09, ns), significant decreases in Cx43 protein are not observed until after more prolonged exposure to 231 CM. Quantitative PCR analysis demonstrates that exposure to 231 CM results in Cx43 mRNA decrease that is evident within 4 h (down to 56.9+/−5.5 % control) and continues to decrease through 12 h (down to 28.4+/−4.0 %) compared to mock media, suggesting that the loss of Cx43 protein is mediated in part by decrease in mRNA (Fig. 3e).

### Functional Cx43-containing GJ are required for mural cell-induced endothelial growth inhibition

**Fig. 4:**
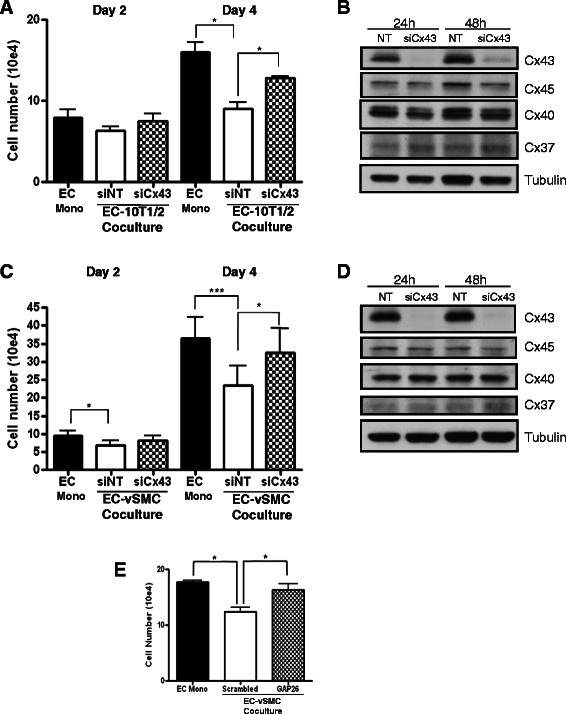
Mural cell inhibition of endothelial cell proliferation requires mural cell Cx43 and GJ communication. Labeled mural cells (**a** and **b**, C3H 10 T1/2 cells, or **c** and **d**, vSMC) nucleofected with control non-targeting or Cx43 siRNA were cultured with labeled HUVEC and effects on HUVEC proliferation assessed by cell counting followed by flow cytometry at indicated days. **a**. Pooled data from three independent experiments performed in triplicate using HUVEC from two different donors. (*p < 0.05). **b**. Western analysis of Cx43 expression from representative experiment from A. **c**. Pooled data from five independent experiments performed in triplicate using vSMC from three different donors and HUVEC from two different donors (*p < 0.05; *** *p* = 0.001). **d**. Western analysis of Cx43 expression from representative experiment from C. **e**. Labeled mural cells were cultured with GFP-HUVEC for four days in the presence of GAP26 blocking peptide or control scrambled peptide and effects on HUVEC proliferation assessed as in A. Data shown are from a representative of two experiment performed in triplicate (*p < 0.05)

We next addressed the functional significance of Cx43 loss in mediating mural cell-endothelial cell growth inhibitory interactions. Cx43 has been shown to be required for endothelial cell contact-induced differentiation of mesenchymal mural precursor cells [9]. Consistent with previous reports [17], the proliferation of endothelial cells in co-culture with C3H10T1/2 mural precursor cells was significantly reduced compared to endothelial cells plated alone (down to 56.5+/−8.3 %, p < 0.05, Fig. 4a); knockdown of Cx43 in C3H10T1/2 cells by siRNA significantly reverses this inhibition of endothelial proliferation (Fig. 4b). We next addressed whether Cx43 expression is similarly required for the endothelial growth inhibition exerted by fully differentiated vSMC cells. While differentiated vSMC exhibit significant growth inhibition on endothelial cells (63.0+/−14.4 %, p < 0.005, Fig. 4c), siRNA knockdown of Cx43 similarly attenuates the ability of vSMC to inhibit endothelial proliferation, indicating a requirement for mural cell Cx43 in this function. Western blot analysis shows Cx43 levels were significantly reduced following siRNA treatment while other connexins remain virtually unaltered (Fig. 4b, d). These data suggest that the observed requirement for Cx43 in mural cell-mediated endothelial inhibition is a general phenomenon.

Since Cx43 can reportedly confer anti-proliferative effects that are independent of its ability to form GJ [22], we sought to determine whether the observed reversal of mural cell-mediated endothelial growth inhibition was dependent on GJ activity. To distinguish this, the co-culture assay was performed in the presence of the blocking peptide Cx43 GAP26 which blocks the formation of Cx43 gap junction channels between cells (reviewed in [23]) but does not affect Cx43 protein levels. In the presence of a scrambled control peptide, vSMC cells retain the ability to inhibit endothelial proliferation (decrease of 30+/−4.5 % compared to EC mono, p < 0.05); in contrast, the Cx43 GAP26 blocking peptide nearly completely reversed the ability of vSMC to inhibit endothelial growth (EC mono 17.7+/−0.3, GAP26 Co 16.3+/− 1.2, ns; Fig. 4e). The Cx43 GAP26 peptide had no significant effect on proliferation of endothelial cells alone (Additional file 2: Figure S2). Together these data indicate that mural cell-mediated endothelial growth inhibition requires gap junction activity that is dependent on mural cell Cx43.

### Breast tumor cell release of endothelial cells from mural cell inhibition is dependent on downregulation of Cx43

**Fig. 5:**
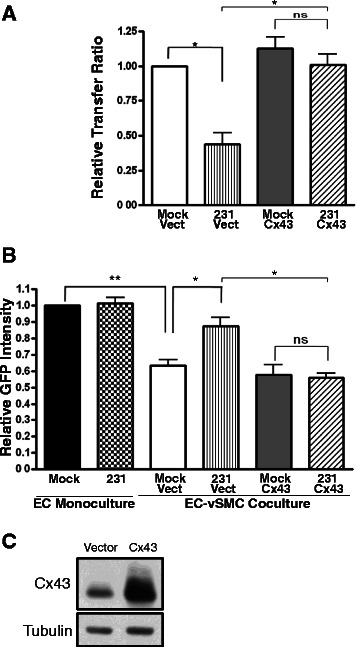
Breast tumor cells inhibit GJ activity and release endothelial cells from mural cell-induced growth inhibition in a Cx43-dependent manner. **a**. vSMC nucleofected with control vector or vector overexpressing wild-type Cx43 were treated with control media (‘Mock’) or MDA-MB-231 conditioned media (‘231 CM’), loaded with calcein-AM, and plated onto HUVEC labeled with PHK26. Dye transfer from vSMC to HUVEC was quantified using flow cytometry (*p < 0.05; ns, not significant). Data represent the mean+/− s.e.m. of three experiments performed in triplicate. **b**. vSMC nucleofected with control vector or vector overexpressing wild-type Cx43 were plated with GFP-HUVEC in the presence of control media or media conditioned for 24 h by 231 cells. Fluorescence was analyzed after four days as in Fig. 1. Data represent the mean+/−s.e.m. of four experiments with two vSMC donors performed in triplicate or duplicate.(*p < 0.05; **p < 0.01; ns, not significant). **c**. Western analysis of vSMC nucleofected with control vector or Cx43 overexpression vector and exposed to MDA-MB-231 conditioned media for 24 h

In order to determine whether loss of Cx43 alone is responsible for the release of endothelial inhibition, we tested whether overexpression of Cx43 was sufficient to restore mural cell growth inhibition of endothelial cells. Although ectopic Cx43 expression does not increase basal GJ dye transfer between mural cells and endothelial cells, mural cells nucleofected with a wild-type Cx43 expression plasmid retain functional gap junctions with endothelial cells despite the presence of tumor media (Fig. 5a; Cx43 Mock vs. Cx43 231, *p* = 0.908, ns). The ability of tumor cells to prevent mural cell-mediated endothelial growth inhibition in co-culture was reversed when mural cells maintain expression of Cx43 (Cx43 231 Co vs. 231 Vector Co, *p* = 0.014; Fig. 5b). Together, these data suggest that tumor-induced loss of heterocellular Cx43 gap junctions promotes endothelial proliferation by releasing endothelial cells from mural cell growth inhibition.

### Proliferation of tumor endothelium is increased and pericyte investiture decreased on a Cx43-reduced host background

**Fig. 6:**
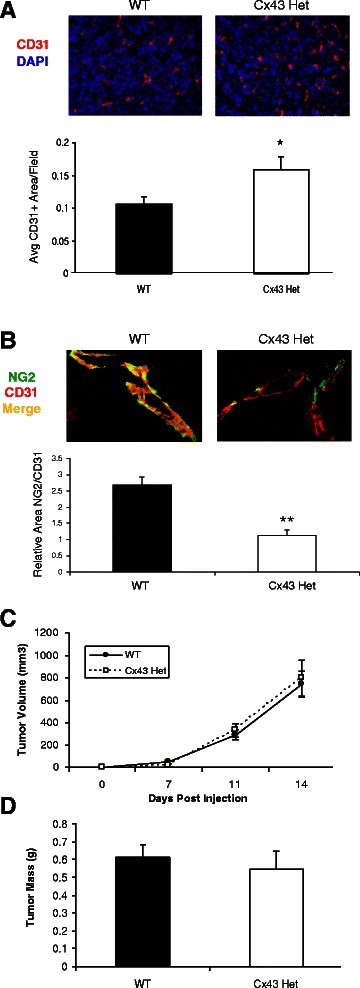
Cx43 deficiency results in enhanced angiogenesis and decreased stabilization but does not affect tumor growth. Eo771 cells were implanted into the mammary fat pad of female mice that were wild-type (‘WT’) or heterozygous (‘Cx43’) for Cx43. **a**. Representative tumor sections stained for CD31 (red) shown at 20x. Bottom panel represents quantification of average CD31+ area per field (*n* = 8 tumors/group). *p < 0.05. **b**. Representative tumor sections stained for NG2 (green) and CD31 (red). Bottom panel represents quantification of relative area of NG2 positivity of CD31 positive vessels (5 fields/tumor, *n* = 3 tumors per group); **p < 0.01. **c**. Tumors were measured using calipers and volume estimated using the formula (((L + W)/2)^3)/2. **d**. Tumor mass was determined at the time of sacrifice (*n* = 10-11)

In order to address the *in vivo* significance of Cx43 loss on angiogenesis, Eo771 mouse mammary tumor cells were implanted into the mammary fat pad of female syngeneic C57Bl/6 wild-type (WT) mice or mice that were bred to be heterozygous for Cx43 (Cx43 Het). Excised tumors from these mice were immunostained for the endothelial marker CD31. Representative images of this staining demonstrate increased CD31-positive (CD31+) endothelial cells in the tumors from Cx43 Het mice compared to those from WT mice (Fig. 6a, top panel). Quantification of the average area of CD31+ endothelial cells confirmed a significant 47.7 % increase in area of CD31+ cells (Fig. 6b lower panel, *p* = 0.023) in Cx43 Het mice compared to WT mice. The presence of NG2+ pericytes on these vessels (Fig. 6b, top panel) was significantly decreased over two-fold in the Cx43 Het mice compared to WT mice (Fig. 6b, lower panel, p < 0.005), suggesting decreased vessel stabilization. Despite the observed increase in vascularity, no change in the growth rate over time (Fig. 6c) or final tumor mass (Fig. 6d) was observed in tumors from Cx43 Het mice compared to WT.

## Discussion

The ability of pericytes to prevent endothelial proliferation is well documented [1, 17, 18], and the vasculature of angiogenic pathologies such as inflammatory arthritis [24], diabetic retinopathy [25], and cancer [4, 5] are characterized by decreased pericyte association with endothelium. The importance of the initial dissociation step in tumor angiogenesis is underscored by the ability of breast cancer cells to increase initiation of angiogenesis without increasing neovessel growth rate [26]. Here we have used an *in vitro* model system to examine the ability of tumors to free endothelial cells from mural cell-induced quiescence in order to mimic this initiation. Mechanistically, we have determined that aggressive tumors may target mural cell Cx43 to prevent or disrupt these functional inhibitory associations to allow for a proliferative and angiogenic endothelium. Our data further indicate a previously unappreciated requirement for mural cell Cx43 expression and gap junction activity in mediating the functional interactions required for maintenance of endothelium in a quiescent state. Since enforced mural cell coverage may decrease angiogenesis [27, 28] and enhance drug delivery [29], strategies to prevent mural Cx43 loss may prove therapeutically beneficial.

Although Cx43 was previously shown to be required for the contact-dependent endothelial cell-induced differentiation of mural cell precursors into functional mural cells [9], our data extend these findings to demonstrate that continued presence of these junctions in already differentiated mural cells is required for maintenance of endothelial quiescence. While several growth factor families including PDGF, Angiopoietin-1, sphingosine-1-phosphate, and TGF-β have been implicated in the recruitment of mural cells during the ‘termination phase’ of normal angiogenesis (reviewed in [30]), the mechanisms by which tumors preclude functional inhibitory interactions between mural cells and endothelial cells have remained ill-defined. Our data suggest that downregulation of mural cell Cx43 by tumors may represent a pivotal point in the initiation of angiogenesis, and/or the ability of tumors to impede the tight functional mural cell-endothelial cell interactions which would render a newly formed vessel mature and quiescent. While the ability to downregulate mural cell Cx43 is restricted to highly aggressive MDA-MB-231 and MDA-MB-468 cell lines in our study, we cannot preclude the possibility that other tumor cells may functionally inactivate Cx43 gap junctions by other means such as phosphorylation or altered Cx43 localization, which can be regulated by different mechanisms. Indeed, it has been demonstrated that breast tumor cells such as MCF7 can recruit mural cells away from vessels *ex vivo* [31], and inactivating phosphorylation of Cx43 on tumor capillaries has been reported [32]. These data are consistent with the possibility that tumor-induced loss of Cx43 gap junction function may diminish the ability of mural cells to associate appropriately with the underlying endothelium, even when they are physically present in the microenvironment.

A connection between Cx43/gap junction decrease and pericyte dissociation has been suggested in other angiogenic pathologies such as diabetic retinopathy, a condition characterized by loss of pericytes from retinal vessels. Previous studies demonstrated that high glucose levels downregulate Cx43 on mural cells [33] and downregulate Cx43 on endothelial cells [34] *in vitro*. Consistent with these findings, a decrease in GJ-mediated dye transfer between retinal pericytes and endothelial cells [35] and a decrease in Cx43 protein in retinal lysates [25] is observed in hyperglycemic rodent models of diabetes. Of note, global Cx43 heterozygous mice exhibit retinal pericyte loss similar to that observed in the diabetic model [25], although a mechanistic link between Cx43/GJIC and lack of proper pericyte association on tumor vasculature has not previously been reported. Similarly, conditions such as ischemia or hypoxia cause pericyte dissociation from brain microvessels *in vivo* [36, 37], and uncouples GJIC in *ex vivo* retinal microvessels [38]. Furthermore, endothelial-specific loss of the TGF-β signaling mediator Smad4 results in vascular defects including impaired association between endothelial cells and pericytes and loss of endothelial Cx43 [39]. Together with our findings, these data from pathologic and genetic models are consistent with a model in which Cx43 loss in one or both vascular cell compartments results in functional pericyte dissociation from endothelium as a consequence of loss of heterocellular GJ communication.

Previous studies have demonstrated that expression of mural Cx43 is required for the endothelial cell-induced differentiation of mural cell precursors into functional, mature mural cells [9]. Since we find that tumors downregulate mural cell Cx43, our data may therefore suggest an explanation for the decreased expression of mature differentiation markers in tumor vessel-associated pericytes [4]. An alternative explanation for decreased mural cell stabilization of tumor vessels is that decreased Cx43 in mural precursors may impair their ability to migrate, as Cx43 is required for the adhesion and motility of several cell types [40]. Additional studies will be required to confirm whether the tumor-induced loss of Cx43 in mural cells contributes to other aspects of vessel defects that are observed in tumors. Interestingly, it has recently been proposed that loss of GJ communication along the vascular wall could result in the arterio-venous shunting defect that is commonly found in tumor vasculature [41]. To our knowledge, our data provide the first empirical evidence that tumors can functionally disrupt vascular GJ activity in support of this hypothesis.

To confirm a requirement for Cx43 in maintaining vessel quiescence *in vivo*, we analyzed tumor angiogenesis in Cx43 heterozygous mice with reduced Cx43 levels. In this experiment, we observed an increase in vascularity as assessed by CD31+ endothelial cells in hosts with reduced Cx43 (Fig. 6a, b). Surprisingly, the observed increase in vascularity in the Cx43 Het mice was not associated with an overall increase in tumor size or growth rate of the Eo771 implanted tumors. It is unclear why tumor growth rate in this model is not increased despite increased vascularity, but possible reasons include that angiogenesis in a wild-type host is not rate-limiting for the highly aggressive Eo771 tumor, or that the excess vessels generated in Cx43-deficient animals may be inefficient or not well-perfused and therefore not provide a proliferative advantage for the tumor. Regardless of the explanation, the increased angiogenesis in Cx43-deficient animals is consistent with our *in vitro* observations that mural cells lacking sufficient Cx43 cannot exert growth inhibitory influence on endothelial cells. Because we used a global Cx43 heterozygote as host, however, we cannot preclude the possibility that Cx43 loss in endothelial cells or host cells other than mural cells may cause or allow increased endothelial proliferation. In contrast with this possibility, previous work suggests that Cx43 heterozygosity is associated with apoptosis of retinal endothelial cells [25], and knockdown of Cx43 in endothelial cells [42] or endothelial progenitor cells impairs proliferation [43]. To better define the role of mural cell Cx43 in tumor vessels, we generated smooth muscle-specific Cx43 knockout mice to use as a tumor host for syngeneic tumor cells. However, we found that a cross between SM-22α-KI mice [44] with Cx43-floxed mice [15] results in neonatal lethality of homozygotes (postnatal day 8–12, unpublished observation). The phenotype of these mice is currently being characterized.

## Conclusions

Our studies identify a novel role for mural cell Cx43 in maintaining quiescence and stability of the vasculature, and suggest that targeting of this molecule by tumors may play a role in the diminished functional mural cell-endothelial association in tumors that allows tumor endothelium to become and remain proliferative. Mural cell Cx43 and vascular GJ communication may therefore prove to be attractive targets to prevent tumor angiogenesis and/or facilitate vessel normalization.

## Additional files

Additional file 1: Figure S1. Conditioned media from MDA-MB-231 cells does not negatively impact SMC proliferation. vSMC plated in monoculture were subjected to MTS assay four days post-plating. (n = 3 experiments performed in triplicate; p < 0.05).
Additional file 2: Figure S2. GAP26 Peptide does not inhibit endothelial proliferation. GFP-HUVEC monocultures were treated for four days in the presence of GAP26 blocking peptide or control scrambled peptide (Scr), then proliferation assessed by cell counting followed by flow cytometry. Data represent mean of three experiments performed in duplicate or triplicate.

## Abbreviations

C: Celsius
Cx: Connexin
CM: Conditioned media
EC: Endothelial cells
ECL: Enhanced chemiluminesence
GFP: Green fluorescent protein
GJ: Gap junction
GJIC: Gap junction intercellular communication
HBMEC: Human brain microvascular endothelial cells
HBVP: Human brain vascular pericytes
Het: Heterozygous
HUVEC: Human umbilical vein endothelial cells
NG2: Neuron-glial chondroitin sulphate proteoglycan antigen 2
NIH: National Institutes of Health
OCT: Optimal cutting temperature compound
vSMC: Vascular smooth muscle cells
WT: Wild-type
