# Supplementary material for: Tumor-induced loss of mural Connexin 43 gap junction activity promotes endothelial proliferation
Source: BMC Cancer. 2015 May 23;15:427. doi: 10.1186/s12885-015-1420-9 (PMC4464240; doi:10.1186/s12885-015-1420-9)
Supplement: Additional file 1: Figure S1. — Conditioned media from MDA-MB-231 cells does not negatively impact SMC proliferation. vSMC plated in monoculture were subjected to MTS assay four days post-plating. (n = 3 experiments performed in triplicate; p < 0.05). [file 12885_2015_1420_MOESM1_ESM.docx]

*

**Supplemental Figure 1. Conditioned media from MDA-MB-231 cells does not negatively impact SMC proliferation.** vSMC plated in monoculture were subjected to MTS assay four days post-plating. (n=3 experiments performed in triplicate; p<0.05)
