# Supplementary material for: Tumor-induced loss of mural Connexin 43 gap junction activity promotes endothelial proliferation
Source: BMC Cancer. 2015 May 23;15:427. doi: 10.1186/s12885-015-1420-9 (PMC4464240; doi:10.1186/s12885-015-1420-9)
Supplement: Additional file 2: Figure S2. — GAP26 Peptide does not inhibit endothelial proliferation. GFP-HUVEC monocultures were treated for four days in the presence of GAP26 blocking peptide or control scrambled peptide (Scr), then proliferation assessed by cell counting followed by flow cytometry. Data represent mean of three experiments performed in duplicate or triplicate. [file 12885_2015_1420_MOESM2_ESM.docx]

**Supplemental File 2. GAP26 Peptide does not inhibit endothelial proliferation.** GFP-HUVEC monocultures were treated for four days in the presence of GAP26 blocking peptide or control scrambled peptide (Scr), then proliferation assessed by cell counting followed by flow cytometry. Data represent mean of three experiments performed in duplicate or triplicate.
